# [μ-1,6-Bis(diphenyphosphan­yl)hexane-1:2κ^2^ *P*:*P*′]deca­carbonyl-1κ^3^ *C*,2κ^3^ *C*,3κ^4^ *C*-*triangulo*-triruthenium(0)

**DOI:** 10.1107/S1600536812014614

**Published:** 2012-04-18

**Authors:** Omar bin Shawkataly, Siti Syaida Sirat, Ching Kheng Quah, Hoong-Kun Fun

**Affiliations:** a Chemical Sciences Programme, School of Distance Education, Universiti Sains Malaysia, 11800 USM, Penang, Malaysia; b X-ray Crystallography Unit, School of Physics, Universiti Sains Malaysia, 11800 USM, Penang, Malaysia

## Abstract

The title *triangulo*-triruthenium(0) compound, [Ru_3_(C_30_H_32_P_2_)(CO)_10_], contains a triangle of singly bonded Ru atoms. The phosphane-bridged Ru—Ru distance [2.9531 (2) Å] is significantly longer than the non-bridged Ru—Ru distances [2.8842 (2) and 2.8876 (2) Å] . The bis­(diphenyl­phosphan­yl)hexane ligand bridges the Ru—Ru bond. Each phosphane-substituted Ru atom bears one equatorial and two axial terminal carbonyl ligands, whereas the unsubstituted Ru atom bears two equatorial and two axial terminal carbonyl ligands. The dihedral angles between the benzene rings attached to each P atom are 72.75 (7) and 82.02 (7)°. The mol­ecular structure is stabilized by an intra­molecular C—H⋯O hydrogen bond involving a methyl­ene group of the phosphane ligand and an axial carbonyl O atom, which generates an *S*(6) ring motif. In the crystal, mol­ecules are linked *via* C—H⋯O hydrogen bonds into layers parallel to (100).

## Related literature

For general background to *triangulo*-triruthenium clusters with structures of the general type Ru_3_(CO)_10_ *L* (where *L* is a group 15 bidentate ligand), see: Bruce *et al.* (1982 ▶); Coleman *et al.* (1984 ▶); Teoh *et al.* (1990 ▶); Diz *et al.* (2001 ▶); Shawkataly *et al.* (2006 ▶, 2011 ▶); Churchill *et al.* (1977 ▶). For the preparation of the title compound, see: Bruce *et al.* (1983 ▶). For hydrogen-bond motifs, see: Bernstein *et al.* (1995 ▶). For the stability of the temperature controller used in the data collection, see: Cosier & Glazer (1986 ▶).
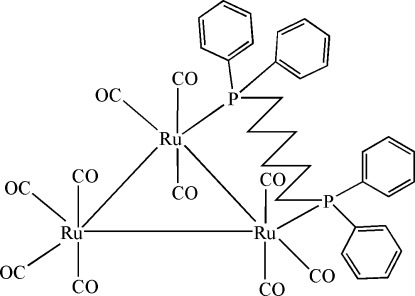


## Experimental

### 

#### Crystal data

[Ru_3_(C_30_H_32_P_2_)(CO)_10_]
*M* *_r_* = 1037.81
Monoclinic, 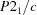

*a* = 13.4836 (6) Å
*b* = 21.270 (1) Å
*c* = 16.1025 (6) Å
β = 122.295 (3)°
*V* = 3903.7 (3) Å^3^
*Z* = 4
Mo *K*α radiation
μ = 1.29 mm^−1^
*T* = 100 K
0.51 × 0.26 × 0.11 mm

#### Data collection

Bruker APEXII DUO CCD area-detector diffractometer
Absorption correction: multi-scan (*SADABS*; Bruker, 2009 ▶) *T*_min_ = 0.562, *T*_max_ = 0.869
53262 measured reflections
14125 independent reflections
12773 reflections with *I* > 2σ(*I*)
*R*_int_ = 0.023

#### Refinement

*R*[*F* ^2^ > 2σ(*F* ^2^)] = 0.019
*wR*(*F* ^2^) = 0.046
*S* = 1.04
14125 reflections
496 parameters
H-atom parameters constrained
Δρ_max_ = 0.54 e Å^−3^
Δρ_min_ = −0.58 e Å^−3^

### 

Data collection: *APEX2* (Bruker, 2009 ▶); cell refinement: *SAINT* (Bruker, 2009 ▶); data reduction: *SAINT*; program(s) used to solve structure: *SHELXTL* (Sheldrick, 2008 ▶); program(s) used to refine structure: *SHELXTL*; molecular graphics: *SHELXTL*; software used to prepare material for publication: *SHELXTL* and *PLATON* (Spek, 2009 ▶).

## Supplementary Material

Crystal structure: contains datablock(s) global, I. DOI: 10.1107/S1600536812014614/sj5228sup1.cif

Structure factors: contains datablock(s) I. DOI: 10.1107/S1600536812014614/sj5228Isup2.hkl

Additional supplementary materials:  crystallographic information; 3D view; checkCIF report

## Figures and Tables

**Table 1:** Hydrogen-bond geometry (Å, °)

*D*—H⋯*A*	*D*—H	H⋯*A*	*D*⋯*A*	*D*—H⋯*A*
C9—H9*A*⋯O3^i^	0.93	2.48	3.3421 (17)	154
C14—H14*A*⋯O6	0.97	2.58	3.2007 (19)	122
C20—H20*A*⋯O6^i^	0.93	2.59	3.495 (2)	164
C21—H21*A*⋯O9^ii^	0.93	2.51	3.329 (2)	147

Symmetry codes: (i) 
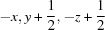
; (ii) 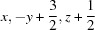.

**Fig. 1.**
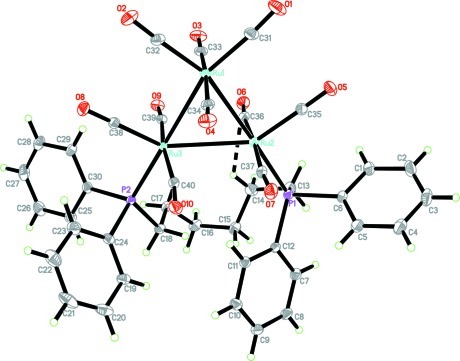
The molecular structure of the title compound showing 40% probability displacement ellipsoids for non-H atoms. Intramolecular hydrogen bond is shown as dash line.

**Fig. 2.**
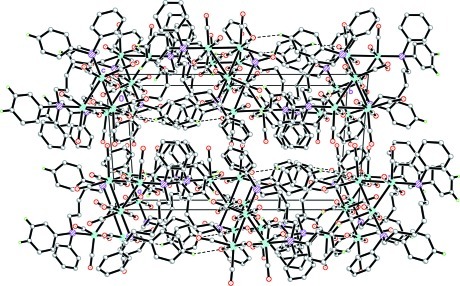
The crystal structure of the title compound, viewed along the c axis. H atoms not involved in hydrogen bonds (dashed lines) have been omitted for clarity.

## supplementary crystallographic information

### Comment

Synthesis and structural reports on substitutued triangulo-triruthenium clusters with group 15 ligands are of interest because of the observed structural variations and their potential catalytic activity. There are several reports of substituted derivatives, of the type Ru_3_(CO)_10_(*L*)[where *L*= bidentate phosphine ligand] (Coleman *et al.*, 1984; Bruce *et al.*, 1982; Teoh *et al.*, 1990; Diz *et al.*, 2001; Shawkataly *et al.*, 2006, 2011).

The title triangulo-triruthenium(0) compound, [Ru_3_(CO)_10_(Ph_2_P(CH_2_)_6_PPh_2_)], contains triangle of singly bonded Ru atoms. These type of structures are derived from that of [Ru_3_(CO)_12_] (Churchill *et al.*, 1977) by replacement of an equatorial carbonyl group on each of two Ru atoms by the Ph_2_P groups of the diphosphane ligands. The phosphane bridged Ru-Ru distance [Ru2—Ru3 = 2.9531 (2) Å ] is significantly longer than the non-bridged Ru-Ru distances [Ru1 —Ru2 = 2.8842 (2) Å and Ru1 — Ru3 = 2.8876 (2) Å]. The bis(diphenylphosphanyl) hexane ligand bridges the Ru2– Ru3 bond. These Ru-Ru distances 2.8842 (2), 2.8876 (2) and 2.9531 (2) Å agree well with those observed in Ru_3_(CO)_10_Ph_2_P(CH_2_)_2_PPh_2_ (2.847 (1), 2.855 (1) and 2.856 (1) Å) (Bruce *et al.*, 1982) andRu_3_(CO)_10_(F-dppe) [where F-dppe = bis(perfluoro-diphenylphosphanyl)ethane] (2.842 (4), 2.849 (4) and 2.868 (4) Å) (Diz *et al.*, 2001) whereby the longest Ru-Ru bond is bridged by the bidentate phosphane ligand. The Ru1 atom carries two equatorial and two axial terminal carbonyl ligands whereas the Ru2 and Ru3 atoms each carries one equatorial and two axial terminal carbonyl ligands. The dihedral angles between the two benzene rings (C1–C6/C7–C12 and C19–C24/C25–C30) are 72.75 (7) and 82.02 (7)°, respectively. The molecular structure is stabilized by an intramolecular C14–H14A···O6 (Table 1) hydrogen bond, which generates an S(6) ring motif (Fig. 1, Bernstein *et al.*, 1995).

In the crystal structure, Fig. 2, molecules are linked via intermolecular C9–H9A···O3, C20–H20A···O6 and C21–H21A···O9 hydrogen bonds (Table 1) into two-dimensional planes parallel to (100).

### Experimental

All manipulations were performed under a dry, oxygen-free nitrogen atmosphere using standard Schlenk techniques. Tetrahydrofuran was dried over sodium and distilled from sodium benzophenone ketyl under nitrogen. The Ru_3_(CO)_12_ (Aldrich) and Ph_2_P(CH_2_)_6_PPh_2_ (Strem Chemicals) were used as received. Ru_3_(CO)_10_(Ph_2_P(CH_2_)_6_PPh_2_) was prepared by a reported procedure (Bruce *et al.*, 1983). The title compound was obtained by reacting equimolar quantities of Ru_3_(CO)_12_ with Ph_2_P(CH_2_)_6_PPh_2_ in 25 ml THF. Crystals suitable for X-ray diffraction were grown by slow solvent/solvent diffusion of CH_3_OH into CH_2_Cl_2_.

### Refinement

All H atoms were positioned geometrically and refined using a riding model with C–H = 0.93 or 0.97 Å and *U*_iso_(H) = 1.2 *U*_eq_(C).

### Crystal data

**Table:**

[Ru_3_(C_30_H_32_P_2_)(CO)_10_]	*F*(000) = 2056
*M**_r_* = 1037.81	*D*_x_ = 1.766 Mg m^−^^3^
Monoclinic, *P*2_1_/*c*	Mo *K*α radiation, λ = 0.71073 Å
Hall symbol: -P 2ybc	Cell parameters from 9965 reflections
*a* = 13.4836 (6) Å	θ = 3.1–32.6°
*b* = 21.270 (1) Å	µ = 1.29 mm^−^^1^
*c* = 16.1025 (6) Å	*T* = 100 K
β = 122.295 (3)°	Block, brown
*V* = 3903.7 (3) Å^3^	0.51 × 0.26 × 0.11 mm
*Z* = 4	

### Data collection

**Table:**

Bruker APEXII DUO CCD area-detector diffractometer	14125 independent reflections
Radiation source: fine-focus sealed tube	12773 reflections with *I* > 2σ(*I*)
Graphite monochromator	*R*_int_ = 0.023
φ and ω scans	θ_max_ = 32.7°, θ_min_ = 1.9°
Absorption correction: multi-scan (*SADABS*; Bruker, 2009)	*h* = −20→20
*T*_min_ = 0.562, *T*_max_ = 0.869	*k* = −15→32
53262 measured reflections	*l* = −24→24

### Refinement

**Table:**

Refinement on *F*^2^	Primary atom site location: structure-invariant direct methods
Least-squares matrix: full	Secondary atom site location: difference Fourier map
*R*[*F*^2^ > 2σ(*F*^2^)] = 0.019	Hydrogen site location: inferred from neighbouring sites
*wR*(*F*^2^) = 0.046	H-atom parameters constrained
*S* = 1.04	*w* = 1/[σ^2^(*F*_o_^2^) + (0.0182*P*)^2^ + 1.6109*P*] where *P* = (*F*_o_^2^ + 2*F*_c_^2^)/3
14125 reflections	(Δ/σ)_max_ = 0.004
496 parameters	Δρ_max_ = 0.54 e Å^−^^3^
0 restraints	Δρ_min_ = −0.58 e Å^−^^3^

### Special details

**Table:**

Experimental. The crystal was placed in the cold stream of an Oxford Cryosystems Cobra open-flow nitrogen cryostat (Cosier & Glazer, 1986) operating at 100.0 (1) K.
Geometry. All esds (except the esd in the dihedral angle between two l.s. planes) are estimated using the full covariance matrix. The cell esds are taken into account individually in the estimation of esds in distances, angles and torsion angles; correlations between esds in cell parameters are only used when they are defined by crystal symmetry. An approximate (isotropic) treatment of cell esds is used for estimating esds involving l.s. planes.
Refinement. Refinement of F^2^ against ALL reflections. The weighted R-factor wR and goodness of fit S are based on F^2^, conventional R-factors R are based on F, with F set to zero for negative F^2^. The threshold expression of F^2^ > 2sigma(F^2^) is used only for calculating R-factors(gt) etc. and is not relevant to the choice of reflections for refinement. R-factors based on F^2^ are statistically about twice as large as those based on F, and R- factors based on ALL data will be even larger.

### Fractional atomic coordinates and isotropic or equivalent isotropic displacement parameters (Å^2^)

**Table:**

	*x*	*y*	*z*	*U*_iso_*/*U*_eq_	
Ru1	0.234282 (8)	0.486493 (4)	0.439613 (7)	0.01568 (2)	
Ru2	0.007592 (7)	0.536230 (4)	0.293260 (7)	0.01454 (2)	
Ru3	0.221591 (7)	0.613182 (4)	0.370118 (6)	0.01222 (2)	
P1	−0.17243 (2)	0.578706 (14)	0.16853 (2)	0.01642 (5)	
P2	0.21229 (2)	0.713604 (13)	0.30678 (2)	0.01351 (5)	
O1	0.16758 (11)	0.35305 (5)	0.46383 (10)	0.0385 (3)	
O2	0.49388 (9)	0.47616 (6)	0.59809 (8)	0.0305 (2)	
O3	0.28204 (10)	0.43668 (5)	0.28450 (8)	0.0287 (2)	
O4	0.19513 (9)	0.54598 (5)	0.59416 (7)	0.0278 (2)	
O5	−0.08834 (9)	0.40860 (5)	0.29939 (8)	0.0266 (2)	
O6	0.05342 (9)	0.48931 (5)	0.13661 (7)	0.02588 (19)	
O7	−0.05743 (9)	0.58536 (5)	0.43750 (7)	0.0266 (2)	
O8	0.47322 (8)	0.62400 (5)	0.54079 (7)	0.02603 (19)	
O9	0.27197 (8)	0.56475 (4)	0.21625 (7)	0.02217 (17)	
O10	0.13472 (8)	0.67573 (4)	0.49164 (7)	0.02337 (18)	
C1	−0.33183 (12)	0.47903 (7)	0.12075 (11)	0.0274 (3)	
H1A	−0.2955	0.4605	0.0915	0.033*	
C2	−0.42138 (13)	0.44728 (7)	0.12156 (12)	0.0338 (3)	
H2A	−0.4448	0.4078	0.0926	0.041*	
C3	−0.47589 (12)	0.47432 (8)	0.16546 (12)	0.0327 (3)	
H3A	−0.5356	0.4529	0.1661	0.039*	
C4	−0.44125 (12)	0.53312 (8)	0.20821 (11)	0.0293 (3)	
H4A	−0.4780	0.5514	0.2373	0.035*	
C5	−0.35137 (11)	0.56491 (7)	0.20765 (10)	0.0237 (2)	
H5A	−0.3279	0.6043	0.2371	0.028*	
C6	−0.29594 (10)	0.53843 (6)	0.16344 (9)	0.0198 (2)	
C7	−0.32163 (10)	0.68222 (6)	0.08869 (9)	0.0218 (2)	
H7A	−0.3698	0.6547	0.0379	0.026*	
C8	−0.35592 (11)	0.74400 (6)	0.08411 (10)	0.0229 (2)	
H8A	−0.4253	0.7583	0.0293	0.027*	
C9	−0.28679 (11)	0.78458 (6)	0.16140 (10)	0.0224 (2)	
H9A	−0.3105	0.8260	0.1590	0.027*	
C10	−0.18240 (11)	0.76352 (6)	0.24221 (9)	0.0215 (2)	
H10A	−0.1366	0.7907	0.2942	0.026*	
C11	−0.14562 (10)	0.70156 (6)	0.24601 (9)	0.0177 (2)	
H11A	−0.0748	0.6879	0.3000	0.021*	
C12	−0.21474 (9)	0.66040 (5)	0.16923 (8)	0.01632 (19)	
C13	−0.20318 (11)	0.56961 (6)	0.04288 (9)	0.0227 (2)	
H13A	−0.2003	0.5252	0.0304	0.027*	
H13B	−0.2823	0.5842	−0.0034	0.027*	
C14	−0.11829 (13)	0.60517 (7)	0.02351 (11)	0.0276 (3)	
H14A	−0.0394	0.5994	0.0801	0.033*	
H14B	−0.1212	0.5856	−0.0322	0.033*	
C15	−0.13912 (11)	0.67564 (7)	0.00301 (9)	0.0232 (2)	
H15A	−0.1500	0.6949	0.0522	0.028*	
H15B	−0.2105	0.6820	−0.0607	0.028*	
C16	−0.03673 (11)	0.70793 (7)	0.00415 (9)	0.0231 (2)	
H16A	−0.0293	0.6900	−0.0476	0.028*	
H16B	−0.0552	0.7522	−0.0106	0.028*	
C17	0.08159 (10)	0.70214 (6)	0.10166 (9)	0.0194 (2)	
H17A	0.1005	0.6580	0.1169	0.023*	
H17B	0.1421	0.7209	0.0944	0.023*	
C18	0.08221 (10)	0.73398 (6)	0.18724 (8)	0.0172 (2)	
H18A	0.0124	0.7215	0.1864	0.021*	
H18B	0.0795	0.7792	0.1785	0.021*	
C19	0.12991 (12)	0.81696 (6)	0.36458 (10)	0.0215 (2)	
H19A	0.0584	0.8121	0.3055	0.026*	
C20	0.14418 (14)	0.86478 (6)	0.42961 (11)	0.0289 (3)	
H20A	0.0820	0.8917	0.4135	0.035*	
C21	0.24985 (16)	0.87246 (7)	0.51764 (11)	0.0324 (3)	
H21A	0.2591	0.9048	0.5601	0.039*	
C22	0.34173 (15)	0.83192 (7)	0.54228 (11)	0.0326 (3)	
H22A	0.4129	0.8368	0.6017	0.039*	
C23	0.32780 (12)	0.78409 (7)	0.47855 (10)	0.0263 (3)	
H23A	0.3895	0.7565	0.4962	0.032*	
C24	0.22258 (10)	0.77667 (5)	0.38820 (9)	0.0170 (2)	
C25	0.32775 (12)	0.79391 (6)	0.24798 (10)	0.0244 (2)	
H25A	0.2639	0.8203	0.2284	0.029*	
C26	0.41702 (13)	0.81267 (7)	0.23448 (11)	0.0294 (3)	
H26A	0.4117	0.8510	0.2045	0.035*	
C27	0.51388 (13)	0.77433 (8)	0.26570 (11)	0.0299 (3)	
H27A	0.5740	0.7872	0.2575	0.036*	
C28	0.52104 (11)	0.71679 (7)	0.30923 (10)	0.0267 (3)	
H28A	0.5864	0.6913	0.3307	0.032*	
C29	0.43030 (10)	0.69707 (6)	0.32084 (9)	0.0203 (2)	
H29A	0.4347	0.6581	0.3487	0.024*	
C30	0.33307 (10)	0.73567 (6)	0.29084 (9)	0.0172 (2)	
C31	0.18777 (12)	0.40334 (6)	0.45353 (11)	0.0252 (3)	
C32	0.39740 (11)	0.48128 (6)	0.53746 (10)	0.0212 (2)	
C33	0.26107 (11)	0.45749 (6)	0.33856 (10)	0.0217 (2)	
C34	0.20400 (11)	0.52583 (6)	0.53304 (10)	0.0208 (2)	
C35	−0.05373 (11)	0.45692 (6)	0.29581 (9)	0.0201 (2)	
C36	0.04399 (10)	0.50930 (6)	0.19821 (10)	0.0203 (2)	
C37	−0.02635 (10)	0.56878 (6)	0.38771 (9)	0.0189 (2)	
C38	0.37863 (10)	0.61975 (5)	0.47485 (9)	0.0177 (2)	
C39	0.24923 (10)	0.57992 (5)	0.27257 (9)	0.0168 (2)	
C40	0.16108 (10)	0.64892 (5)	0.44471 (9)	0.0170 (2)	

### Atomic displacement parameters (Å^2^)

**Table:**

	*U*^11^	*U*^22^	*U*^33^	*U*^12^	*U*^13^	*U*^23^
Ru1	0.01584 (4)	0.01339 (4)	0.02068 (4)	0.00335 (3)	0.01167 (3)	0.00438 (3)
Ru2	0.01199 (4)	0.01355 (4)	0.01822 (4)	−0.00056 (3)	0.00816 (3)	−0.00232 (3)
Ru3	0.01112 (4)	0.01189 (4)	0.01314 (4)	0.00063 (3)	0.00614 (3)	0.00121 (3)
P1	0.01275 (11)	0.01649 (12)	0.01796 (13)	−0.00106 (9)	0.00683 (10)	−0.00405 (10)
P2	0.01276 (11)	0.01292 (11)	0.01512 (12)	0.00080 (9)	0.00762 (10)	0.00159 (9)
O1	0.0567 (7)	0.0181 (4)	0.0655 (8)	0.0020 (5)	0.0492 (7)	0.0052 (5)
O2	0.0213 (4)	0.0415 (6)	0.0276 (5)	0.0099 (4)	0.0124 (4)	0.0101 (4)
O3	0.0399 (5)	0.0225 (4)	0.0327 (5)	0.0099 (4)	0.0254 (5)	0.0058 (4)
O4	0.0331 (5)	0.0297 (5)	0.0264 (5)	0.0078 (4)	0.0197 (4)	0.0051 (4)
O5	0.0330 (5)	0.0193 (4)	0.0354 (5)	−0.0065 (4)	0.0235 (4)	−0.0062 (4)
O6	0.0247 (4)	0.0281 (5)	0.0280 (5)	−0.0006 (4)	0.0163 (4)	−0.0075 (4)
O7	0.0257 (4)	0.0307 (5)	0.0302 (5)	−0.0051 (4)	0.0195 (4)	−0.0077 (4)
O8	0.0165 (4)	0.0280 (5)	0.0237 (4)	−0.0011 (3)	0.0041 (3)	0.0029 (4)
O9	0.0284 (4)	0.0205 (4)	0.0219 (4)	0.0029 (3)	0.0162 (4)	0.0022 (3)
O10	0.0276 (4)	0.0204 (4)	0.0293 (5)	−0.0031 (3)	0.0200 (4)	−0.0045 (4)
C1	0.0203 (5)	0.0245 (6)	0.0328 (7)	−0.0057 (5)	0.0110 (5)	−0.0068 (5)
C2	0.0249 (6)	0.0293 (7)	0.0364 (8)	−0.0113 (5)	0.0091 (6)	−0.0031 (6)
C3	0.0169 (5)	0.0404 (8)	0.0320 (7)	−0.0055 (5)	0.0071 (5)	0.0094 (6)
C4	0.0207 (6)	0.0371 (7)	0.0312 (7)	0.0030 (5)	0.0146 (5)	0.0097 (6)
C5	0.0203 (5)	0.0249 (6)	0.0263 (6)	0.0009 (4)	0.0127 (5)	0.0021 (5)
C6	0.0129 (4)	0.0204 (5)	0.0225 (5)	−0.0009 (4)	0.0071 (4)	−0.0007 (4)
C7	0.0151 (5)	0.0237 (5)	0.0205 (5)	0.0001 (4)	0.0054 (4)	−0.0020 (5)
C8	0.0159 (5)	0.0252 (6)	0.0238 (6)	0.0045 (4)	0.0082 (4)	0.0051 (5)
C9	0.0219 (5)	0.0189 (5)	0.0283 (6)	0.0035 (4)	0.0147 (5)	0.0033 (5)
C10	0.0228 (5)	0.0183 (5)	0.0223 (5)	−0.0006 (4)	0.0112 (5)	−0.0032 (4)
C11	0.0157 (5)	0.0187 (5)	0.0167 (5)	0.0003 (4)	0.0073 (4)	−0.0011 (4)
C12	0.0138 (4)	0.0169 (5)	0.0174 (5)	0.0004 (4)	0.0077 (4)	−0.0015 (4)
C13	0.0215 (5)	0.0253 (6)	0.0201 (5)	−0.0037 (4)	0.0102 (5)	−0.0075 (5)
C14	0.0314 (7)	0.0268 (6)	0.0312 (7)	−0.0001 (5)	0.0212 (6)	−0.0033 (5)
C15	0.0183 (5)	0.0305 (6)	0.0167 (5)	0.0021 (5)	0.0067 (4)	0.0030 (5)
C16	0.0210 (5)	0.0313 (6)	0.0147 (5)	0.0041 (5)	0.0080 (4)	0.0049 (5)
C17	0.0177 (5)	0.0247 (5)	0.0158 (5)	0.0024 (4)	0.0089 (4)	0.0019 (4)
C18	0.0160 (5)	0.0188 (5)	0.0162 (5)	0.0028 (4)	0.0082 (4)	0.0026 (4)
C19	0.0272 (6)	0.0168 (5)	0.0267 (6)	0.0036 (4)	0.0186 (5)	0.0034 (4)
C20	0.0468 (8)	0.0183 (5)	0.0377 (7)	0.0062 (5)	0.0333 (7)	0.0035 (5)
C21	0.0619 (10)	0.0191 (6)	0.0292 (7)	−0.0046 (6)	0.0331 (7)	−0.0038 (5)
C22	0.0443 (8)	0.0274 (7)	0.0223 (6)	−0.0061 (6)	0.0152 (6)	−0.0059 (5)
C23	0.0269 (6)	0.0243 (6)	0.0217 (6)	0.0002 (5)	0.0090 (5)	−0.0031 (5)
C24	0.0208 (5)	0.0140 (4)	0.0189 (5)	−0.0004 (4)	0.0123 (4)	0.0007 (4)
C25	0.0263 (6)	0.0203 (5)	0.0320 (7)	−0.0015 (4)	0.0192 (5)	0.0029 (5)
C26	0.0352 (7)	0.0259 (6)	0.0363 (7)	−0.0090 (5)	0.0252 (6)	0.0001 (6)
C27	0.0265 (6)	0.0389 (8)	0.0334 (7)	−0.0117 (6)	0.0221 (6)	−0.0068 (6)
C28	0.0179 (5)	0.0369 (7)	0.0292 (6)	−0.0018 (5)	0.0151 (5)	−0.0036 (6)
C29	0.0164 (5)	0.0248 (5)	0.0213 (5)	−0.0001 (4)	0.0111 (4)	0.0004 (4)
C30	0.0170 (5)	0.0178 (5)	0.0188 (5)	−0.0024 (4)	0.0110 (4)	−0.0008 (4)
C31	0.0302 (6)	0.0196 (5)	0.0371 (7)	0.0043 (5)	0.0255 (6)	0.0035 (5)
C32	0.0226 (5)	0.0222 (5)	0.0243 (6)	0.0064 (4)	0.0162 (5)	0.0071 (5)
C33	0.0240 (5)	0.0163 (5)	0.0269 (6)	0.0053 (4)	0.0150 (5)	0.0062 (4)
C34	0.0193 (5)	0.0190 (5)	0.0256 (6)	0.0045 (4)	0.0129 (5)	0.0066 (4)
C35	0.0198 (5)	0.0186 (5)	0.0246 (6)	−0.0009 (4)	0.0138 (5)	−0.0048 (4)
C36	0.0159 (5)	0.0198 (5)	0.0251 (6)	−0.0004 (4)	0.0109 (4)	−0.0025 (4)
C37	0.0147 (4)	0.0186 (5)	0.0221 (5)	−0.0024 (4)	0.0089 (4)	−0.0025 (4)
C38	0.0174 (5)	0.0168 (5)	0.0191 (5)	0.0006 (4)	0.0099 (4)	0.0023 (4)
C39	0.0173 (5)	0.0140 (4)	0.0177 (5)	0.0013 (4)	0.0085 (4)	0.0030 (4)
C40	0.0159 (4)	0.0155 (5)	0.0196 (5)	−0.0012 (4)	0.0095 (4)	0.0018 (4)

### Geometric parameters (Å, º)

**Table:**

Ru1—C32	1.9050 (13)	C8—H8A	0.9300
Ru1—C31	1.9293 (14)	C9—C10	1.3849 (18)
Ru1—C34	1.9459 (13)	C9—H9A	0.9300
Ru1—C33	1.9461 (14)	C10—C11	1.3980 (17)
Ru1—Ru2	2.8842 (2)	C10—H10A	0.9300
Ru1—Ru3	2.8876 (2)	C11—C12	1.3899 (16)
Ru2—C35	1.8887 (13)	C11—H11A	0.9300
Ru2—C36	1.9271 (13)	C13—C14	1.5366 (19)
Ru2—C37	1.9354 (13)	C13—H13A	0.9700
Ru2—P1	2.3522 (3)	C13—H13B	0.9700
Ru2—Ru3	2.9531 (2)	C14—C15	1.528 (2)
Ru3—C38	1.8792 (12)	C14—H14A	0.9700
Ru3—C39	1.9319 (12)	C14—H14B	0.9700
Ru3—C40	1.9322 (12)	C15—C16	1.5333 (19)
Ru3—P2	2.3413 (3)	C15—H15A	0.9700
P1—C12	1.8308 (12)	C15—H15B	0.9700
P1—C6	1.8353 (12)	C16—C17	1.5326 (17)
P1—C13	1.8458 (13)	C16—H16A	0.9700
P2—C24	1.8285 (12)	C16—H16B	0.9700
P2—C18	1.8334 (11)	C17—C18	1.5315 (17)
P2—C30	1.8399 (12)	C17—H17A	0.9700
O1—C31	1.1376 (17)	C17—H17B	0.9700
O2—C32	1.1402 (16)	C18—H18A	0.9700
O3—C33	1.1387 (16)	C18—H18B	0.9700
O4—C34	1.1368 (16)	C19—C24	1.3902 (17)
O5—C35	1.1429 (15)	C19—C20	1.3974 (19)
O6—C36	1.1472 (16)	C19—H19A	0.9300
O7—C37	1.1409 (16)	C20—C21	1.381 (2)
O8—C38	1.1468 (15)	C20—H20A	0.9300
O9—C39	1.1480 (15)	C21—C22	1.382 (2)
O10—C40	1.1445 (15)	C21—H21A	0.9300
C1—C2	1.390 (2)	C22—C23	1.385 (2)
C1—C6	1.3954 (18)	C22—H22A	0.9300
C1—H1A	0.9300	C23—C24	1.3959 (17)
C2—C3	1.388 (2)	C23—H23A	0.9300
C2—H2A	0.9300	C25—C26	1.3910 (19)
C3—C4	1.383 (2)	C25—C30	1.4012 (17)
C3—H3A	0.9300	C25—H25A	0.9300
C4—C5	1.3920 (19)	C26—C27	1.386 (2)
C4—H4A	0.9300	C26—H26A	0.9300
C5—C6	1.3969 (18)	C27—C28	1.388 (2)
C5—H5A	0.9300	C27—H27A	0.9300
C7—C8	1.3818 (18)	C28—C29	1.3973 (17)
C7—C12	1.4061 (16)	C28—H28A	0.9300
C7—H7A	0.9300	C29—C30	1.3961 (17)
C8—C9	1.3869 (19)	C29—H29A	0.9300
			
C32—Ru1—C31	98.85 (6)	C12—C11—H11A	120.0
C32—Ru1—C34	90.69 (5)	C10—C11—H11A	120.0
C31—Ru1—C34	95.10 (6)	C11—C12—C7	118.84 (11)
C32—Ru1—C33	91.44 (5)	C11—C12—P1	122.69 (9)
C31—Ru1—C33	91.35 (6)	C7—C12—P1	118.47 (9)
C34—Ru1—C33	172.82 (5)	C14—C13—P1	114.55 (9)
C32—Ru1—Ru2	161.50 (4)	C14—C13—H13A	108.6
C31—Ru1—Ru2	99.37 (4)	P1—C13—H13A	108.6
C34—Ru1—Ru2	84.58 (4)	C14—C13—H13B	108.6
C33—Ru1—Ru2	91.27 (4)	P1—C13—H13B	108.6
C32—Ru1—Ru3	100.25 (4)	H13A—C13—H13B	107.6
C31—Ru1—Ru3	160.89 (4)	C15—C14—C13	117.11 (11)
C34—Ru1—Ru3	84.54 (4)	C15—C14—H14A	108.0
C33—Ru1—Ru3	88.33 (4)	C13—C14—H14A	108.0
Ru2—Ru1—Ru3	61.547 (3)	C15—C14—H14B	108.0
C35—Ru2—C36	92.98 (5)	C13—C14—H14B	108.0
C35—Ru2—C37	90.71 (5)	H14A—C14—H14B	107.3
C36—Ru2—C37	176.27 (5)	C14—C15—C16	112.10 (11)
C35—Ru2—P1	95.60 (4)	C14—C15—H15A	109.2
C36—Ru2—P1	91.18 (4)	C16—C15—H15A	109.2
C37—Ru2—P1	87.95 (4)	C14—C15—H15B	109.2
C35—Ru2—Ru1	86.55 (4)	C16—C15—H15B	109.2
C36—Ru2—Ru1	86.07 (4)	H15A—C15—H15B	107.9
C37—Ru2—Ru1	94.67 (4)	C17—C16—C15	114.47 (10)
P1—Ru2—Ru1	176.602 (9)	C17—C16—H16A	108.6
C35—Ru2—Ru3	145.79 (4)	C15—C16—H16A	108.6
C36—Ru2—Ru3	83.75 (4)	C17—C16—H16B	108.6
C37—Ru2—Ru3	93.48 (3)	C15—C16—H16B	108.6
P1—Ru2—Ru3	118.456 (9)	H16A—C16—H16B	107.6
Ru1—Ru2—Ru3	59.283 (4)	C18—C17—C16	112.87 (10)
C38—Ru3—C39	98.20 (5)	C18—C17—H17A	109.0
C38—Ru3—C40	93.46 (5)	C16—C17—H17A	109.0
C39—Ru3—C40	168.09 (5)	C18—C17—H17B	109.0
C38—Ru3—P2	95.48 (4)	C16—C17—H17B	109.0
C39—Ru3—P2	88.20 (3)	H17A—C17—H17B	107.8
C40—Ru3—P2	88.26 (3)	C17—C18—P2	112.48 (8)
C38—Ru3—Ru1	85.21 (4)	C17—C18—H18A	109.1
C39—Ru3—Ru1	88.63 (3)	P2—C18—H18A	109.1
C40—Ru3—Ru1	94.79 (3)	C17—C18—H18B	109.1
P2—Ru3—Ru1	176.823 (8)	P2—C18—H18B	109.1
C38—Ru3—Ru2	142.89 (4)	H18A—C18—H18B	107.8
C39—Ru3—Ru2	91.49 (3)	C24—C19—C20	119.99 (13)
C40—Ru3—Ru2	80.56 (3)	C24—C19—H19A	120.0
P2—Ru3—Ru2	120.673 (8)	C20—C19—H19A	120.0
Ru1—Ru3—Ru2	59.170 (4)	C21—C20—C19	120.57 (13)
C12—P1—C6	99.50 (5)	C21—C20—H20A	119.7
C12—P1—C13	102.59 (6)	C19—C20—H20A	119.7
C6—P1—C13	103.60 (6)	C20—C21—C22	119.71 (13)
C12—P1—Ru2	122.83 (4)	C20—C21—H21A	120.1
C6—P1—Ru2	110.90 (4)	C22—C21—H21A	120.1
C13—P1—Ru2	114.87 (4)	C21—C22—C23	119.98 (14)
C24—P2—C18	104.06 (5)	C21—C22—H22A	120.0
C24—P2—C30	100.32 (5)	C23—C22—H22A	120.0
C18—P2—C30	102.40 (5)	C22—C23—C24	121.02 (13)
C24—P2—Ru3	113.02 (4)	C22—C23—H23A	119.5
C18—P2—Ru3	118.14 (4)	C24—C23—H23A	119.5
C30—P2—Ru3	116.62 (4)	C19—C24—C23	118.68 (12)
C2—C1—C6	120.65 (14)	C19—C24—P2	122.90 (9)
C2—C1—H1A	119.7	C23—C24—P2	118.41 (9)
C6—C1—H1A	119.7	C26—C25—C30	120.65 (13)
C3—C2—C1	120.18 (14)	C26—C25—H25A	119.7
C3—C2—H2A	119.9	C30—C25—H25A	119.7
C1—C2—H2A	119.9	C27—C26—C25	120.05 (13)
C4—C3—C2	119.89 (13)	C27—C26—H26A	120.0
C4—C3—H3A	120.1	C25—C26—H26A	120.0
C2—C3—H3A	120.1	C26—C27—C28	119.99 (12)
C3—C4—C5	119.99 (14)	C26—C27—H27A	120.0
C3—C4—H4A	120.0	C28—C27—H27A	120.0
C5—C4—H4A	120.0	C27—C28—C29	120.18 (13)
C4—C5—C6	120.80 (13)	C27—C28—H28A	119.9
C4—C5—H5A	119.6	C29—C28—H28A	119.9
C6—C5—H5A	119.6	C30—C29—C28	120.31 (12)
C1—C6—C5	118.49 (12)	C30—C29—H29A	119.8
C1—C6—P1	120.81 (10)	C28—C29—H29A	119.8
C5—C6—P1	120.58 (10)	C29—C30—C25	118.81 (11)
C8—C7—C12	120.79 (11)	C29—C30—P2	122.63 (9)
C8—C7—H7A	119.6	C25—C30—P2	118.56 (9)
C12—C7—H7A	119.6	O1—C31—Ru1	175.53 (12)
C7—C8—C9	119.91 (11)	O2—C32—Ru1	176.94 (12)
C7—C8—H8A	120.0	O3—C33—Ru1	174.44 (11)
C9—C8—H8A	120.0	O4—C34—Ru1	173.73 (11)
C10—C9—C8	120.02 (12)	O5—C35—Ru2	178.36 (13)
C10—C9—H9A	120.0	O6—C36—Ru2	171.84 (11)
C8—C9—H9A	120.0	O7—C37—Ru2	173.03 (10)
C9—C10—C11	120.31 (12)	O8—C38—Ru3	177.79 (11)
C9—C10—H10A	119.8	O9—C39—Ru3	173.87 (10)
C11—C10—H10A	119.8	O10—C40—Ru3	171.83 (10)
C12—C11—C10	120.08 (11)		
			
C32—Ru1—Ru2—C35	−170.75 (13)	C12—P1—C6—C1	152.39 (11)
C31—Ru1—Ru2—C35	−0.70 (6)	C13—P1—C6—C1	46.84 (12)
C34—Ru1—Ru2—C35	−94.99 (5)	Ru2—P1—C6—C1	−76.90 (12)
C33—Ru1—Ru2—C35	90.88 (5)	C12—P1—C6—C5	−31.60 (11)
Ru3—Ru1—Ru2—C35	178.29 (4)	C13—P1—C6—C5	−137.14 (11)
C32—Ru1—Ru2—C36	96.01 (13)	Ru2—P1—C6—C5	99.11 (10)
C31—Ru1—Ru2—C36	−93.93 (6)	C12—C7—C8—C9	−2.6 (2)
C34—Ru1—Ru2—C36	171.78 (5)	C7—C8—C9—C10	1.2 (2)
C33—Ru1—Ru2—C36	−2.35 (5)	C8—C9—C10—C11	0.6 (2)
Ru3—Ru1—Ru2—C36	85.06 (4)	C9—C10—C11—C12	−1.06 (19)
C32—Ru1—Ru2—C37	−80.33 (13)	C10—C11—C12—C7	−0.26 (18)
C31—Ru1—Ru2—C37	89.73 (6)	C10—C11—C12—P1	179.77 (9)
C34—Ru1—Ru2—C37	−4.56 (5)	C8—C7—C12—C11	2.09 (19)
C33—Ru1—Ru2—C37	−178.69 (5)	C8—C7—C12—P1	−177.93 (10)
Ru3—Ru1—Ru2—C37	−91.28 (4)	C6—P1—C12—C11	119.28 (11)
C32—Ru1—Ru2—P1	59.97 (19)	C13—P1—C12—C11	−134.37 (10)
C31—Ru1—Ru2—P1	−129.98 (15)	Ru2—P1—C12—C11	−3.29 (12)
C34—Ru1—Ru2—P1	135.73 (15)	C6—P1—C12—C7	−60.70 (11)
C33—Ru1—Ru2—P1	−38.40 (15)	C13—P1—C12—C7	45.66 (11)
Ru3—Ru1—Ru2—P1	49.01 (14)	Ru2—P1—C12—C7	176.73 (8)
C32—Ru1—Ru2—Ru3	10.95 (12)	C12—P1—C13—C14	71.06 (11)
C31—Ru1—Ru2—Ru3	−178.99 (4)	C6—P1—C13—C14	174.24 (10)
C34—Ru1—Ru2—Ru3	86.72 (4)	Ru2—P1—C13—C14	−64.66 (11)
C33—Ru1—Ru2—Ru3	−87.41 (4)	P1—C13—C14—C15	−79.49 (14)
C32—Ru1—Ru3—C38	−7.39 (5)	C13—C14—C15—C16	169.24 (11)
C31—Ru1—Ru3—C38	172.15 (13)	C14—C15—C16—C17	−59.62 (15)
C34—Ru1—Ru3—C38	82.32 (5)	C15—C16—C17—C18	−63.05 (15)
C33—Ru1—Ru3—C38	−98.55 (5)	C16—C17—C18—P2	168.47 (9)
Ru2—Ru1—Ru3—C38	169.10 (4)	C24—P2—C18—C17	159.32 (9)
C32—Ru1—Ru3—C39	90.96 (5)	C30—P2—C18—C17	55.19 (10)
C31—Ru1—Ru3—C39	−89.51 (13)	Ru3—P2—C18—C17	−74.45 (9)
C34—Ru1—Ru3—C39	−179.33 (5)	C24—C19—C20—C21	−0.1 (2)
C33—Ru1—Ru3—C39	−0.20 (5)	C19—C20—C21—C22	−1.0 (2)
Ru2—Ru1—Ru3—C39	−92.56 (3)	C20—C21—C22—C23	0.4 (2)
C32—Ru1—Ru3—C40	−100.47 (5)	C21—C22—C23—C24	1.3 (2)
C31—Ru1—Ru3—C40	79.06 (13)	C20—C19—C24—C23	1.69 (18)
C34—Ru1—Ru3—C40	−10.76 (5)	C20—C19—C24—P2	−179.12 (10)
C33—Ru1—Ru3—C40	168.37 (5)	C22—C23—C24—C19	−2.3 (2)
Ru2—Ru1—Ru3—C40	76.02 (3)	C22—C23—C24—P2	178.48 (12)
C32—Ru1—Ru3—P2	95.40 (15)	C18—P2—C24—C19	16.52 (12)
C31—Ru1—Ru3—P2	−85.07 (19)	C30—P2—C24—C19	122.23 (11)
C34—Ru1—Ru3—P2	−174.89 (15)	Ru3—P2—C24—C19	−112.87 (10)
C33—Ru1—Ru3—P2	4.24 (15)	C18—P2—C24—C23	−164.28 (10)
Ru2—Ru1—Ru3—P2	−88.11 (14)	C30—P2—C24—C23	−58.58 (11)
C32—Ru1—Ru3—Ru2	−176.49 (4)	Ru3—P2—C24—C23	66.33 (11)
C31—Ru1—Ru3—Ru2	3.05 (13)	C30—C25—C26—C27	−1.6 (2)
C34—Ru1—Ru3—Ru2	−86.78 (4)	C25—C26—C27—C28	0.9 (2)
C33—Ru1—Ru3—Ru2	92.35 (4)	C26—C27—C28—C29	0.6 (2)
C35—Ru2—Ru3—C38	−21.23 (9)	C27—C28—C29—C30	−1.5 (2)
C36—Ru2—Ru3—C38	−107.34 (7)	C28—C29—C30—C25	0.78 (19)
C37—Ru2—Ru3—C38	75.17 (7)	C28—C29—C30—P2	−178.39 (10)
P1—Ru2—Ru3—C38	164.72 (6)	C26—C25—C30—C29	0.7 (2)
Ru1—Ru2—Ru3—C38	−18.20 (6)	C26—C25—C30—P2	179.95 (11)
C35—Ru2—Ru3—C39	84.48 (8)	C24—P2—C30—C29	118.01 (11)
C36—Ru2—Ru3—C39	−1.62 (5)	C18—P2—C30—C29	−134.96 (10)
C37—Ru2—Ru3—C39	−179.12 (5)	Ru3—P2—C30—C29	−4.39 (12)
P1—Ru2—Ru3—C39	−89.57 (3)	C24—P2—C30—C25	−61.16 (11)
Ru1—Ru2—Ru3—C39	87.51 (3)	C18—P2—C30—C25	45.87 (11)
C35—Ru2—Ru3—C40	−104.44 (8)	Ru3—P2—C30—C25	176.43 (9)
C36—Ru2—Ru3—C40	169.45 (5)	C32—Ru1—C31—O1	−27.5 (18)
C37—Ru2—Ru3—C40	−8.04 (5)	C34—Ru1—C31—O1	−119.0 (18)
P1—Ru2—Ru3—C40	81.51 (4)	C33—Ru1—C31—O1	64.1 (18)
Ru1—Ru2—Ru3—C40	−101.41 (3)	Ru2—Ru1—C31—O1	155.6 (18)
C35—Ru2—Ru3—P2	173.27 (7)	Ru3—Ru1—C31—O1	152.9 (17)
C36—Ru2—Ru3—P2	87.17 (4)	C31—Ru1—C32—O2	−25 (2)
C37—Ru2—Ru3—P2	−90.32 (4)	C34—Ru1—C32—O2	70 (2)
P1—Ru2—Ru3—P2	−0.775 (13)	C33—Ru1—C32—O2	−117 (2)
Ru1—Ru2—Ru3—P2	176.308 (10)	Ru2—Ru1—C32—O2	145 (2)
C35—Ru2—Ru3—Ru1	−3.03 (7)	Ru3—Ru1—C32—O2	155 (2)
C36—Ru2—Ru3—Ru1	−89.14 (4)	C32—Ru1—C33—O3	48.8 (13)
C37—Ru2—Ru3—Ru1	93.37 (4)	C31—Ru1—C33—O3	−50.1 (13)
P1—Ru2—Ru3—Ru1	−177.083 (10)	C34—Ru1—C33—O3	155.9 (11)
C35—Ru2—P1—C12	141.36 (6)	Ru2—Ru1—C33—O3	−149.5 (13)
C36—Ru2—P1—C12	−125.53 (6)	Ru3—Ru1—C33—O3	149.0 (13)
C37—Ru2—P1—C12	50.85 (6)	C32—Ru1—C34—O4	−27.4 (11)
Ru1—Ru2—P1—C12	−89.57 (15)	C31—Ru1—C34—O4	71.6 (11)
Ru3—Ru2—P1—C12	−42.00 (5)	C33—Ru1—C34—O4	−134.6 (10)
C35—Ru2—P1—C6	24.19 (6)	Ru2—Ru1—C34—O4	170.5 (11)
C36—Ru2—P1—C6	117.30 (6)	Ru3—Ru1—C34—O4	−127.6 (11)
C37—Ru2—P1—C6	−66.32 (6)	C36—Ru2—C35—O5	100 (4)
Ru1—Ru2—P1—C6	153.26 (14)	C37—Ru2—C35—O5	−81 (4)
Ru3—Ru2—P1—C6	−159.17 (4)	P1—Ru2—C35—O5	−169 (4)
C35—Ru2—P1—C13	−92.83 (6)	Ru1—Ru2—C35—O5	14 (4)
C36—Ru2—P1—C13	0.28 (6)	Ru3—Ru2—C35—O5	17 (4)
C37—Ru2—P1—C13	176.66 (6)	C35—Ru2—C36—O6	37.9 (8)
Ru1—Ru2—P1—C13	36.24 (16)	C37—Ru2—C36—O6	−134.1 (8)
Ru3—Ru2—P1—C13	83.81 (5)	P1—Ru2—C36—O6	−57.7 (8)
C38—Ru3—P2—C24	−67.34 (6)	Ru1—Ru2—C36—O6	124.3 (8)
C39—Ru3—P2—C24	−165.41 (5)	Ru3—Ru2—C36—O6	−176.2 (8)
C40—Ru3—P2—C24	25.97 (5)	C35—Ru2—C37—O7	−48.1 (10)
Ru1—Ru3—P2—C24	−169.85 (14)	C36—Ru2—C37—O7	123.9 (10)
Ru2—Ru3—P2—C24	103.93 (4)	P1—Ru2—C37—O7	47.4 (10)
C38—Ru3—P2—C18	170.88 (6)	Ru1—Ru2—C37—O7	−134.7 (9)
C39—Ru3—P2—C18	72.82 (6)	Ru3—Ru2—C37—O7	165.8 (9)
C40—Ru3—P2—C18	−95.80 (6)	C39—Ru3—C38—O8	−165 (3)
Ru1—Ru3—P2—C18	68.38 (15)	C40—Ru3—C38—O8	17 (3)
Ru2—Ru3—P2—C18	−17.85 (5)	P2—Ru3—C38—O8	106 (3)
C38—Ru3—P2—C30	48.17 (6)	Ru1—Ru3—C38—O8	−78 (3)
C39—Ru3—P2—C30	−49.90 (5)	Ru2—Ru3—C38—O8	−62 (3)
C40—Ru3—P2—C30	141.48 (5)	C38—Ru3—C39—O9	−55.2 (10)
Ru1—Ru3—P2—C30	−54.34 (16)	C40—Ru3—C39—O9	112.9 (9)
Ru2—Ru3—P2—C30	−140.56 (4)	P2—Ru3—C39—O9	40.1 (10)
C6—C1—C2—C3	−0.3 (2)	Ru1—Ru3—C39—O9	−140.2 (10)
C1—C2—C3—C4	0.2 (2)	Ru2—Ru3—C39—O9	160.7 (10)
C2—C3—C4—C5	−0.4 (2)	C38—Ru3—C40—O10	50.2 (7)
C3—C4—C5—C6	0.6 (2)	C39—Ru3—C40—O10	−118.0 (7)
C2—C1—C6—C5	0.5 (2)	P2—Ru3—C40—O10	−45.2 (7)
C2—C1—C6—P1	176.64 (11)	Ru1—Ru3—C40—O10	135.7 (7)
C4—C5—C6—C1	−0.7 (2)	Ru2—Ru3—C40—O10	−166.7 (7)
C4—C5—C6—P1	−176.80 (10)		

### Hydrogen-bond geometry (Å, º)

**Table:**

*D*—H···*A*	*D*—H	H···*A*	*D*···*A*	*D*—H···*A*
C9—H9*A*···O3^i^	0.93	2.48	3.3421 (17)	154
C14—H14*A*···O6	0.97	2.58	3.2007 (19)	122
C20—H20*A*···O6^i^	0.93	2.59	3.495 (2)	164
C21—H21*A*···O9^ii^	0.93	2.51	3.329 (2)	147

Symmetry codes: (i) −*x*, *y*+1/2, −*z*+1/2; (ii) *x*, −*y*+3/2, *z*+1/2.
